# Height identification of water-permeable fractured zone based on synchronous movement in overlying strata

**DOI:** 10.1038/s41598-022-11752-1

**Published:** 2022-05-09

**Authors:** Zhiqiang Wang, Jingkai Li, Zhongcheng Qin, Yue Su, Shermatova Sayyora Sidikovna

**Affiliations:** 1 School of Energy and Mining Engineering, China University of Mining and Technology (Beijing), Beijing, 100083 China; 2 Russian Academy of Natural Sciences, Moscow, 119991 Russia; 3 College of Energy and Mining Engineering, Shandong University of Science and Technology, Qingdao, 266590 China; 4 China Coal International Engineering Design and Research Institute, Beijing, 100120 China; 5 National University of Science and Technology «MISIS», Moscow, Russia 119049

**Keywords:** Engineering, Civil engineering

## Abstract

Height identification of water-permeable fractured zone (WPFZ) is one of the decisive influence factors for mining safety, especially in some specific conditions, such as mining under aquifer. In order to demonstrate the formation process of the WPFZ, the scaling model experiment is carried out. Through the analysis of movement and breaking in overlying strata, the WPFZ height is significantly affected by mining range, movement characteristics of key strata and its follow-up strata. Based on the research findings, a new theoretical method, " overlying strata synchronous movement method " (OSSM) is established to predict the WPFZ height. Taking 3301 mining face of Zhujiamao Coal Mine in China as the engineering background, the WPFZ height is estimated by OSSM. Additionally, the field detection is carried out by the downhole segmented water injection method combined with borehole camera method. By comparing the results of different methods, the accuracy of OSSM is verified and the WPFZ height is determined finally. What´s more, various methods for determining WPFZ height are evaluated.

## Introduction

**Figure 1:**
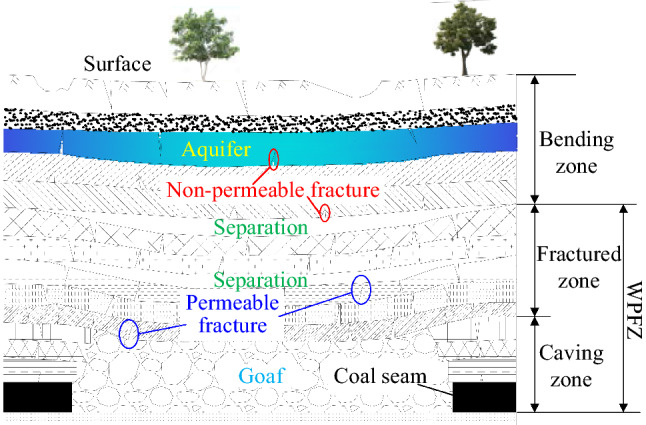
Caving zone, fractured zone and bending zone in the overlying strata after mining (This figure is drawn by the AutoCAD 2018, https://www.autodesk.com.cn/products/autocad/overview?term=1-YEAR&tab=subscription).

In subsurface coal mining, the overburden will be moved, deformed and destroyed after the coal seam is mined^1–3^. As presented in Fig. 1, according to the movement and deformation, the overlying strata are divided into caving zone, fractured zone and bending zone after mining^4, 5^. Caving zone and fractured zone are collectively referred to as WPFZ, in which a large number of mining-induced fractures are developed, which are the main channels for fluid migration and infiltration, so the WPFZ is extremely permeable. If the WPFZ height is greater than or equal to the distance between the coal seam and the aquifer, the aquifer will be destroyed and the water in the aquifer will pour into the mining face or roadway through the WPFZ, resulting in groundwater loss and mine disaster^6, 7^. Therefore, the accurate prediction of WPFZ height is the basic work for determining the mining upper limit, coal mining technology and its parameters. Likewise, the determination of WPFZ height is of great significance for water-preserved-mining^8, 9^.

The distribution of new fractures in overlying strata is very complex after the coal seam is mined out^10, 11^. Therefore, the WPFZ height is affected not only by the thickness of mining coal seam, mining methods, but also by the lithologic characteristics, engineering geological conditions and other factors^12, 13^. In view of these factors, many scholars have made a lot of exploration to predict the WPFZ height. Currently, there are many methods to determine the WPFZ height, such as simulation experiment method, field detection method and theoretical analysis method^14, 15^. Generally, simulation experiment method, that is, simulating the failure height of overburden through numerical analysis or scaling model experiment can provide a basis for determining WPFZ height^16, 17^. Field detection methods mainly include ground borehole flushing fluid method, geophysical exploration method, downhole segmented water injection method and borehole camera method^18, 19^. The ground borehole flushing fluid method determines the WPFZ height by detecting the leakage of borehole flushing fluid during drilling, which has complex structure and low detection accuracy. Geophysical exploration method can determine the WPFZ height by analyzing the variation of resistivity in collapsed and cracked rock mass, but its exploration cost is high and it is easily affected by high temperature, high humidity and other factors. Downhole segmented water injection method is to determine the WPFZ height by detecting the water seepage of upward-inclined boreholes above the mining goaf, and the borehole camera technique is to put the detector into the boreholes to observe the separation of rock mass. The theoretical analysis method is characterized by simplification. Therefore, many theoretical analysis methods have been established^20^.

For various theoretical analysis methods, although the traditional empirical formula method in China is widely applied because of its simplicity, the prediction methods based on key strata is more objective^21–23^. The distance between key strata and coal seam and the thickness of mined coal seam are considered in the existing methods based on key strata. However, we find that the WPFZ height is also significantly affected by mining range, movement characteristics of key strata and its follow-up strata, especially the zoning of follow-up strata after the key strata fracted. On the basis of this finding, the new method of "overlying strata synchronous movement method" is established to predict the WPFZ height. In the new method, the influence of the mined coal seam thickness, the mining range, the residual fragmentation coefficient of overlying strata, the key strata and its follow-up strata movement characteristics on the WPFZ height are considered comprehensively. Therefore, the WPFZ height predicted by this new method is more accurate, which can provide more convenient guidance for coal mines as well.

The field engineering example applied in this paper is 3301 mining face of Zhujiamao Coal Mine in China. The identification of WPFZ height follows a framework that applies several progressive and mutually validated methods. Firstly, the "overlying strata synchronous movement method" is applied to predict the WPFZ height. Secondly, the field detection is carried out by the downhole segmented water injection method combined with borehole camera method to verify the reliability of the new method. These methods verify each other and determine the height of WPFZ comprehensively.

## Establishment of new method

### Review of empirical formulas in China

The empirical formulas included in the “Coal mining regulations under the buildings, water and rails” are widely utilized to predict WPFZ height in China. As presented in Eq. (1) to Eq. (4) ^24^, the empirical formulas only include two factors of coal thickness and lithology. The overlying strata are divided into hard, medium-hard, soft and significantly soft categories according to the comprehensive compressive strength. Furthermore, the WFZ height could be calculated from the mining thickness by the corresponding empirical formulas for different roof lithology^25^. Although this method is simple, many key factors that affect the height of WPFZ are ignored, resulting in inaccurate height. Therefore, more accurate theoretical methods need to be explored.

1 $${\text{For the hard strata category}}:H = \frac{100\Sigma M}{{1.2\Sigma M + 2.0}} \pm 8.9$$

2 $${\text{For the medium hard strata category}}:H = \frac{100\Sigma M}{{1.6\Sigma M + 3.6}} \pm 5.6$$

3 $${\text{For the soft strata category}}:H = \frac{100\Sigma M}{{3.1\Sigma M + 5.0}} \pm 4.0$$

4 $${\text{For the significantly soft strata category}}:H = \frac{100\Sigma M}{{5.0\Sigma M + 8.0}} \pm 3.0$$

where *H* is the WPFZ height and Σ*M* is the accumulated mining thickness.

#### Influence of synchronous movement in overlying strata

**Figure 2:**
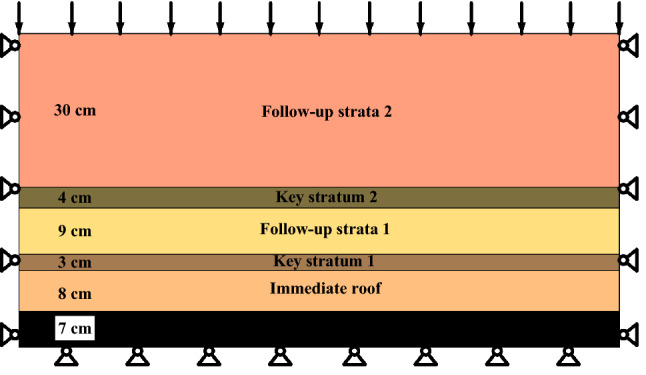
Simplified experimental model.

In order to intuitively reflect the synchronous movement of overlying strata during the mining, that is, the influence of the key strata and its follow-up strata on the WPFZ height, the scaling model experiment is adopted. This experiment is carried out on bench with sizes 120 cm × 8 cm × 100 cm (length × width × height). The objective of the experiment is to study the influence of the key strata and its follow-up strata on the WPFZ. Therefore, the model is simplified by laying the key strata and the follow-up strata alternately, as presented in Fig. 2. According to similarity theory^26–28^, the geometric similarity ratio is 100 and the bulk density similarity ratio is 0.6. The model material takes sand as aggregate, gypsum and lime as binder, and mica is scattered at the junction of rock stratum to separate strata. The material ratio is shown in Table 1.

**Table 1:** Materials ratio.

Stratum	Thickness/cm	Sand/kg	Gypsum/kg	Lime/kg	Water/kg
Follow-up stratum 2	30	12	0.7	1.7	1.4
Key stratum 2	4	4.3	1.0	0.4	0.57
Follow-up stratum 1	9	10.8	0.6	1.5	1.3
Key stratum 1	3	3.2	0.76	0.32	0.43
Immediate roof	8	9.6	0.6	1.34	1.15
Coal seam	7	8.8	0.4	0.9	1.01

**Figure 3:**
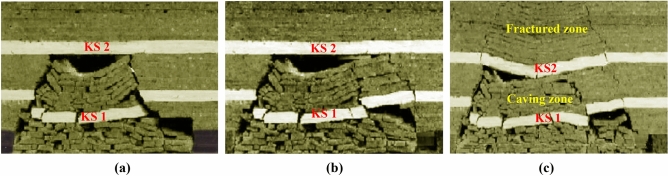
Experimental process: (**a**) advanced to 56 m, (**b**) advanced to 63 m, and (**c**) advanced to 87 m.

The experimental results are presented in Fig. 3, in the figures, “key stratum” is abbreviated as “KS”. As presented in Fig. 3a, when the mining face is advanced to 56 m, the key stratum 1 breaks after the hanging length reaches 35 m. The key stratum 1 collapses into the goaf after fractures due to the large cavity below. Correspondingly, the follow-up strata with a thickness of 9 m collapse with the key stratum 1 synchronously. Therefore, the height of the caving zone increases from 8 m before the fracture of key stratum 1–20 m, and the WPFZ height is 20 m. Additionally, the key stratum 2 doesn´t reach the ultimate fracture length and remains stable.

When the face is advanced to 63 m, the key stratum 2 still remains stable due to the caving angle of overburden, although the fracture length reaches 36 m. Synchronously, the follow-up strata above key stratum 2 remains stable as well. The WPFZ is still within the range of 20 m above the coal seam. This experimental process is presented in Fig. 3b.

As presented in Fig. 3c, when the face is advanced to 87 m, the hanging length of key stratum 2 approaches to 58 m, resulting in its fracture. The key stratum 2 breaks and sinks rotationally to form a hinged structure due to the great fracture length and the small space below. The range of 20 m below the key stratum 2 belongs to the caving zone, the key stratum 2 and its follow-up strata belong to the fractured zone. Simultaneously, the WPFZ is in the range of 34 m above the coal seam.

Due to the size limitation of the experimental bench, the experimental process and results need to be further inferred. Multiple key strata are assumed to be located above the key stratum 2. After the fracture, the key stratum 2 sinks greatly, and the upper follow-up strata moves synchronously, due to the broken rock which is gradually compacted below. Therefore, it will lead to cavities below the higher key strata. With the increase of the mining range, the corresponding key strata will break when they reach the limit hanging lengths. Otherwise, they will remain stable.

As we can see from the experimental results, the stability of the key strata could be affected by the mining space, and the WPFZ height is determined by the synchronous movement of the key strata and its follow-up strata. The height of the caving zone or fractured zone is fixed before the key strata approach to the limit hanging length. However, at the limit hanging length, the key strata break and the follow-up strata above it move synchronously. Whether the key strata can form a hinged structure determines the zone to which the key strata and its follow-up strata belong after fracture. That is, if the key stratum forms a hinged structure, the key stratum and its follow-up strata belong to the fractured zone; on the contrary, the key stratum and its follow-up strata belong to the caving zone.

#### New method to predict WPFZ height

**Figure 4:**
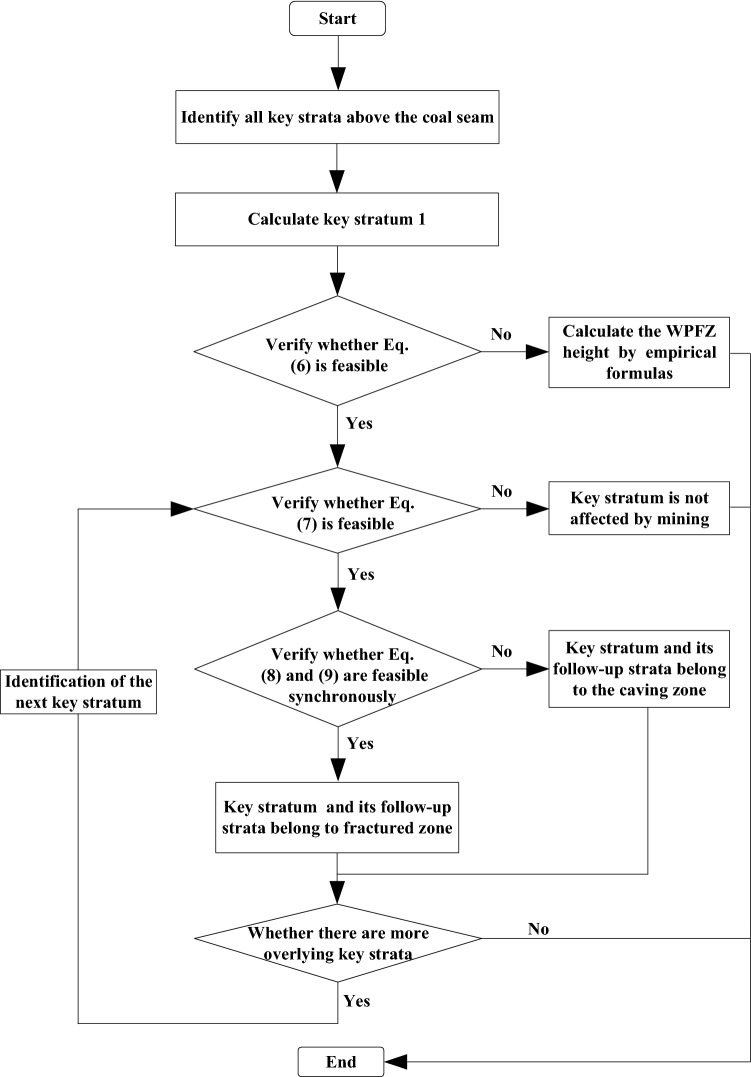
Flow chart of the calculation procedure.

Based on the above experiment and theory, the OSSM is proposed to predict the WPFZ height. As is presented in Fig. 4, the WPFZ height can be determined using the following procedures.

**Figure 5:**
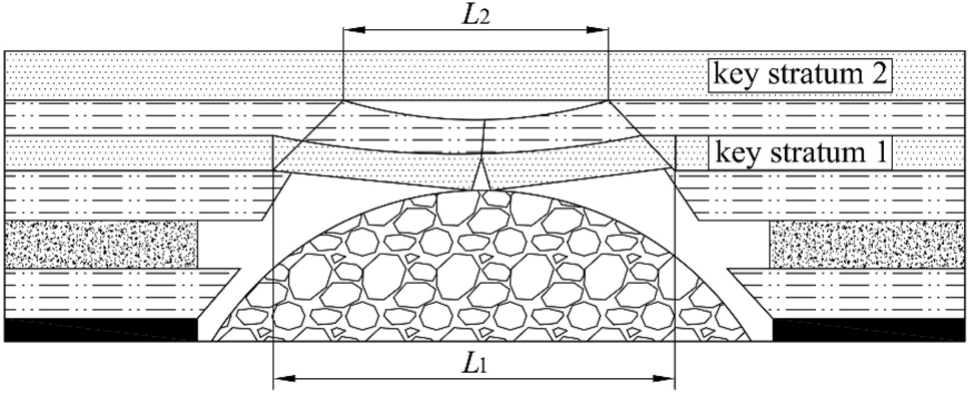
Schematic diagram of key strata breaking (This figure is drawn by the AutoCAD 2018, https://www.autodesk.com.cn/products/autocad/overview?term=1-YEAR&tab=subscription).

Step I: Identify all key strata above the coal seam. For the determination of key strata, we can refer to these perfect research^29^. At the same time, we also need to pay additional attention to the strength conditions of key strata. As presented in Fig. 5, Multiple key strata are assumed to exist. The lower key stratum (key stratum 1) will fracture after it reaches the fracture length *L*_1_, and the fracture develops upward along a certain span angle. If there are still key stratum in the upper strata (key stratum 2), it shall meet the requirements of Eq. (5)^30^.

5 $$\left\{ \begin{gathered} L_{1} = L_{2} + 2\sum\limits_{i = 1}^{n} {h_{i} } \cot \alpha \hfill \\ \tan \alpha = \frac{{\sum\limits_{i = 1}^{n} {h_{i} } }}{{h_{1} \cot \alpha_{1} + h_{2} \cot \alpha_{2} \cdot \cdot \cdot + h_{n} \cot \alpha_{n} }} \hfill \\ \end{gathered} \right.$$

where *L*_1_ is the fracture length of the lower key stratum, *L*_2_ is the overhang length of the upper key stratum when the lower key stratum fractures, h_*i*_ is the thickness of each stratum, *α* is the average fracture angle of overlying strata, *α*_*i*_ is the fracture angle of each stratum.

Step II: Analyze whether there will be cavities under the key stratum 1 during the mining. In this step, the mining height of the face, the distance between the face and the key stratum 1, and the residual fragmentation coefficient of the rock strata below the key stratum 1 are comprehensively considered. That is, verify whether Eq. (6) is feasible^30^.

6 $$h_{r} + M \ge K_{p}^{^{\prime}} \left[ {h_{r} + \left( {1 - C} \right)M} \right]$$

where *h*_*r*_ is the height of immediate roof, *M* is the mining thickness, $K_{p}^{^{\prime}}$ the residual fragmentation coefficient and *C* is the recovery rate.

If Eq. (6) is feasible, there will be cavities under the key stratum 1 when the immediate roof collapses. Otherwise, the WPFZ height should be calculated directly according to the empirical formulas.

Step III: If there are cavities under the key stratum 1, combined with the design mining parameters of the face to analyze whether key stratum 1 will break, namely, whether Eq. (7) is feasible^30^.

7 $$\alpha - 2\Sigma h\cot \alpha \ge h_{1} \left( {{{2R_{t} } \mathord{\left/ {\vphantom {{2R_{t} } q}} \right. \kern-\nulldelimiterspace} q}} \right)^{{{\raise0.7ex\hbox{$1$} \!\mathord{\left/ {\vphantom {1 2}}\right.\kern-\nulldelimiterspace} \!\lower0.7ex\hbox{$2$}}}}$$

where ∑*h* is the cumulative thickness of the strata between the key stratum 1 and the face, *h*_1_ is the thickness of key stratum 1, *R*_t_ is the tensile strength of the key stratum 1 and *q* is load-bearing of the key stratum 1.

If Eq. (7) is feasible, the key stratum 1 will fracture due to the influence of mining range. On the contrary, key stratum 1 is not affected by mining. At the same time, the caving strata below key stratum belong to the caving zone, and only caving zone appears in the whole overlying strata.

Step IV: If the key stratum 1 fractures, it will be necessary to further identify whether it belongs to the caving zone or fractured zone. The basis of identification is based on the principle of "three hinged arch"^31, 32^, that is:

(1) Condition for non-sliding instability of fractured blocks:

8 $${{h_{1} } \mathord{\left/ {\vphantom {{h_{1} } {L_{1} }}} \right. \kern-\nulldelimiterspace} {L_{1} }} \le {1 \mathord{\left/ {\vphantom {1 {2\tan \theta }}} \right. \kern-\nulldelimiterspace} {2\tan \theta }}$$

where *θ* is the friction angle between rock blocks.

(2) Condition for non-deformation instability of fractured blocks:

9 $$\left\{ \begin{gathered} \sigma_{{\text{p}}} /\sigma_{c} \le k \hfill \\ \sigma_{p} = {{2qi^{2} } \mathord{\left/ {\vphantom {{2qi^{2} } {\left( {1 - i\sin \beta } \right)}}} \right. \kern-\nulldelimiterspace} {\left( {1 - i\sin \beta } \right)}}^{2} \hfill \\ \end{gathered} \right.$$

where *σ*_p_ is compressive force at the interlocking areas of fractured rock blocks, $i = {{L_{1} } \mathord{\left/ {\vphantom {{L_{1} } {h_{k} }}} \right. \kern-\nulldelimiterspace} {h_{k} }}$, *σ*_c_ is the compressive strength of rock blocks, *k* is the proportional coefficient determined by experience and *β* is the allowable subsidence angle of rock blocks after fracture.

If the key stratum 1 after fracture satisfies Eq. (8) and Eq. (9) simultaneously, then the key stratum 1 and its follow-up strata belong to fractured zone. On the contrary, they both belong to the caving zone.

Step V: When the key stratum 1 is determined as belonging to the fractured zone after the fracture, steps III to IV need to be repeated to calculate the next stratum (the corresponding parameters in the formula shall be changed to the parameters of the key stratum under calculation). Additionally, when the key stratum 1 is determined as belonging to the caving zone, and there is only one key stratum in the overlying strata, the caving zone extends from the mining face to the surface. Generally, many shallow coal seams present this mine pressure behavior. However, when there are multiple key strata in the overburden, steps III to IV need to be repeated to calculate the next stratum as well. Moreover, there are several situations that need to be considered:

1. One is that the next key stratum does not break within the mining range. In this circumstance, there are only caving zone and bending zone above the mining face, and there is no fractured zone.
2. Another possibility is that the next key stratum breaks within the mining range. Furthermore, if hinged structures can be formed after the fracture, then the key stratum and its follow-up strata belong to the fractured zone. On the contrary, if the hinged structures can´t be formed after the fracture, then the key stratum and the follow-up strata belong to the caving zone. Therefore, steps III to IV to need to be repeated to identify the other key strata above.

#### Application requirements of the new method

The OSSM is easy to calculate, but it has some application requirements. Firstly, the coal seam in the face should be fully mined at one time and the fallen method should be applied to control roof. Secondly, the application of the new method requires a complete grasp of many factors, such as the physical and mechanical properties of the overlying strata and the mining parameters of the mining face.

### Application of the new method

#### Study area

**Figure 6:**
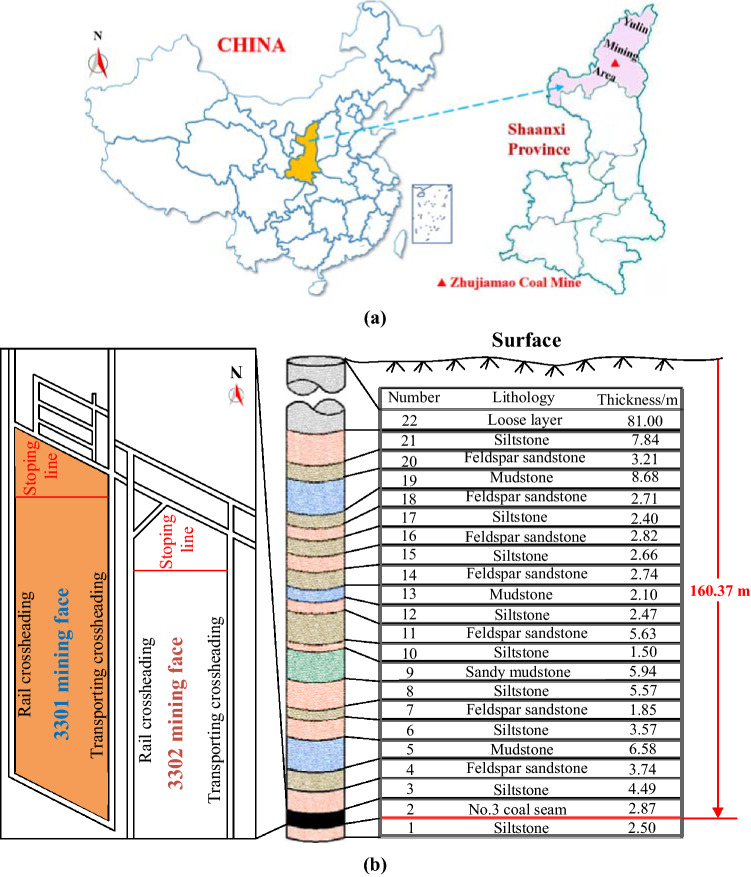
Study site: (**a**) Zhuajiamao mine in Yulin, Shannxi Province, China, and (**b**) Stratigraphic section above the No. 3 coal seam and mining face layout.

As presented in Fig. 6a, Zhujiamao Coal Mine is located in Yulin mining area, Shaanxi Province, China. Yulin mining area is rich in coal resources, with shallow coal seams and simple geological environment, which is conducive to large-scale mining. However, the mining area is located in the transitional zone between the southern edge of Mu Us Desert and the Loess Plateau. Correspondingly, its ecological environment is extremely fragile and vulnerable to destruction. Additionally, the overlying bedrock of the coal seam is thin, and the aquifer in the alluvium is close to the shallow main coal seam. Therefore, unreasonable mining will cause the loss of water resource, mine water gushing-out, and other accidents.

Zhujiamao Coal Mine mainly mines No. 3 coal seam, with average buried depth of 160.37 m, dip angle of approximately horizontal and average thickness of 2.87 m, belonging to medium thick coal seam. The mining face is arranged with strike longwall, the comprehensive mechanized mining face with full-seam mining is adopted, and the roof is controlled with fallen method. As presented in Fig. 6b, the lithologies of coal seam roof are mainly siltstone and feldspar sandstone, followed by mudstone and a small amount of sandy mudstone. The direct water-filled aquifer of coal seam is Jurassic Yan'an Formation, Zhiluo Formation sandstone fractured aquifer and Quaternary aquifer, with medium water-rich property.

In order to study the developing height of WPFZ in overburden of No. 3 coal seam, the exploration is carried out in No.3 panel. 3301 mining face is the first mining face in No. 3 panel. Thus, selecting 3301 mining face as the detection location can determine the WPFZ height after the first mining. Moreover, the risk of roof sandstone aquifer and Quaternary aquifer of No. 3 coal seam can be predicted and evaluated as well. Ultimately, all these findings can provide support for mine water prevention.

#### WPFZ height prediction by the new method

The rock strata of 3301 mining face in Zhujiamao mine is presented in Fig. 6b. According to the criterion for determining key strata, there are two key strata above the mining coal seam, namely, siltstone (key stratum 1) and mudstone (key stratum 2) with numbers of 6 and 19 respectively. The OSSM is applied to calculate the WPFZ height. It can be found that the key stratum 1 breaks during the mining, and the blocks form hinged structures. Therefore, the key stratum 1 and its follow-up strata belong to the range of the fractured zone. Additionally, the rock strata below the key stratum 1 belong to the caving zone with a height of 14.81 m. However, the key stratum 2 is far away from the coal seam, and the collapsed rock mass below it can fill the goaf. Therefore, the key stratum 2 is not hanged and fractured, because it is not affected by mining. And then, the strata below key stratum 2 belong to the range of the fractured zone. Finally, the height of caving zone, fractured zone and WPFZ is identified as 14.81 m, 44.83 m and 59.6 m respectively.

#### Validation of the new method

In order to more accurately determine the WPFZ height in the study area and verify the accuracy of the application of the OSSM, downhole segmented water injection method and borehole camera method are implemented in the field.

##### Downhole segmented water injection method

(1) Detection principle

**Figure 7:**
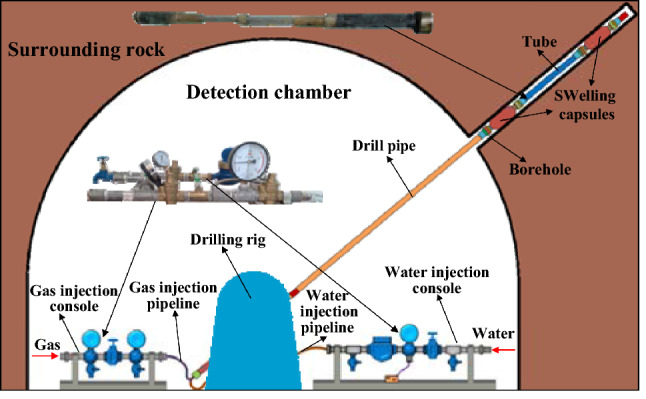
Layout of detection system (This figure is drawn by the Microsoft office 2016, https://www.microsoft.com/zh-cn/microsoft-365/previous-versions/microsoft-office-2016).

Figure 7 presents the layout of the detection system. The detection chamber is located adjacent to the detected mining face, the upward-inclined boreholes are drilled in this chamber towards the overlying strata above the mining goaf. The borehole is divided into several segments, and each segment is sealed separately. The sealed segments are successively injected with water for detection until the top of the borehole. The length of each segment is about 1–2 m. During the detection, the water seepage is various due to the different locations that the probe locates. Therefore, by analyzing the variation of water seepage in each segment, the fracture development and water permeability of rock strata can be judged individually. Based on these detected results, the WPFZ height can be determined comprehensively^33^. The main equipment adopted is the water injection tube. Multiple holes are fabricated along the tube to allow high-pressure water injection into the borehole. Correspondingly, a water injection console is utilized to control the high-pressure water injection. In addition, two connected capsules are set at both ends of the tube simultaneously. The capsules can be pushed to any depth of the borehole because they are contracted in the non-working state. During the detection, these two capsules are filled with gas to seal the detecting segments of the boreholes. Similarly, a gas injection console is utilized as well to control the gas. The tube and the two consoles constitute the probe which is connected by pipelines and drill pipes, and the drilling rig enables the probe to move^25^.

(2) Detection scheme

The goaf of 3301 mining face is selected as the location to detect the WPFZ height. Factors, such as stability of surrounding rock, ventilation, pedestrian, water supply and court size should be taken into account in the selection of detection chamber. Combined with the engineering geological conditions, the detection chamber is arranged in the middle of rail crossheading of 3302 mining face. Three detection boreholes with different coordinate azimuths and dip angles are drilled on the roof of the same detection chamber. As a pre-mining detection borehole, borehole B1 is applied to detect the development of original fractures in overlying strata. For comparisons, boreholes B2 and B3 are applied to detect the development of fractures after mining. By comparing the water seepage in boreholes before and after mining, the WPFZ height can be accurately determined.

**Figure 8:**
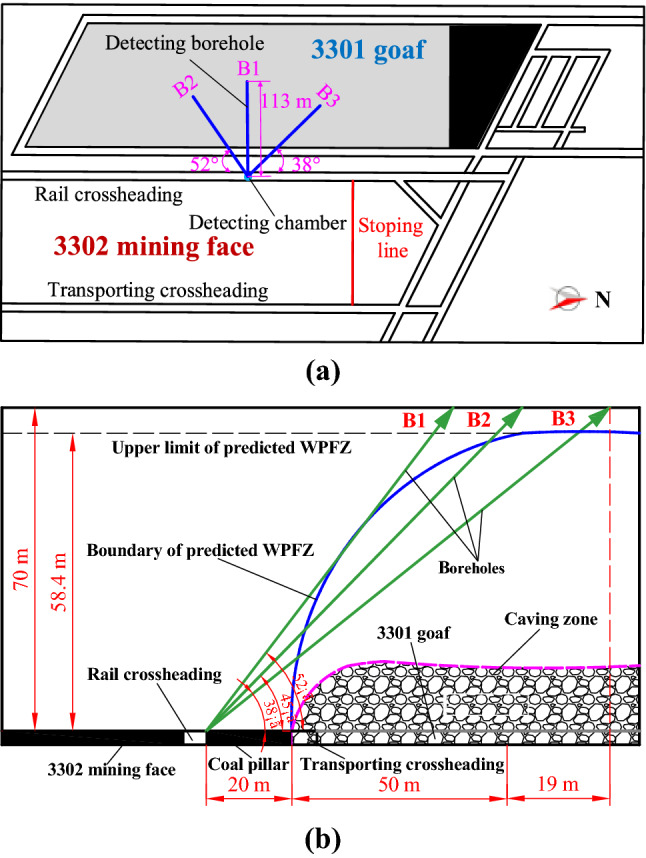
Layout of detection boreholes: (**a**) horizontal projection of detection boreholes, and (**b**) profile of the detection boreholes.

**Figure 9:**
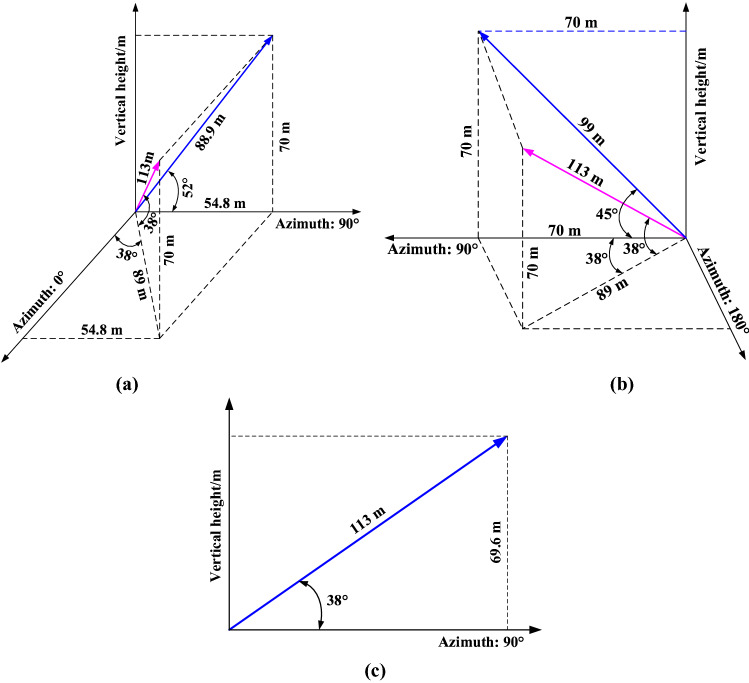
Three-dimensional diagram of detection boreholes: (**a**) Borehole B1, (**b**) Borehole B2 and (**c**) Borehole B3.

The design of borehole parameters is the key to successful detection. Therefore, these following parameters must be satisfied: the dip angle must ensure that the probe can get into the WPFZ; the length must ensure that the probe can pierce through the WPFZ (i.e., the vertical height of borehole must reach the predicted WPFZ height). The WPFZ height determined from the "overlying strata synchronous movement method" is 59.6 m. Considering the possible errors in the calculation, the WPFZ height is predicted to be 70 m, so as to fully guarantee the reliability of field detection. Hence, the lowest vertical heights of the detection boreholes are 70 m. According to the design requirements, the parameters are obtained by mathematical trigonometric calculation, as presented in Table 2. Additionally, the boreholes layout is presented in Fig. 8 and the three-dimensional diagram of boreholes is presented in Fig. 9.

**Table 2:** Design parameters for detection boreholes.

Parameters	Labels		
	B1	B2	B3
Coordinate azimuth/°	38	128	90
Dip angle/°	38	38	38
Length/m	113	113	113
Vertical height/m	70	70	70
Diameter/mm	85	85	85

(3) Detection results

The detection results indicate that the difference between the water seepage in detection boreholes before and after mining is apparent. Furthermore, the water seepage in detection boreholes presents obvious segmented characteristics after mining.

**Figure 10:**
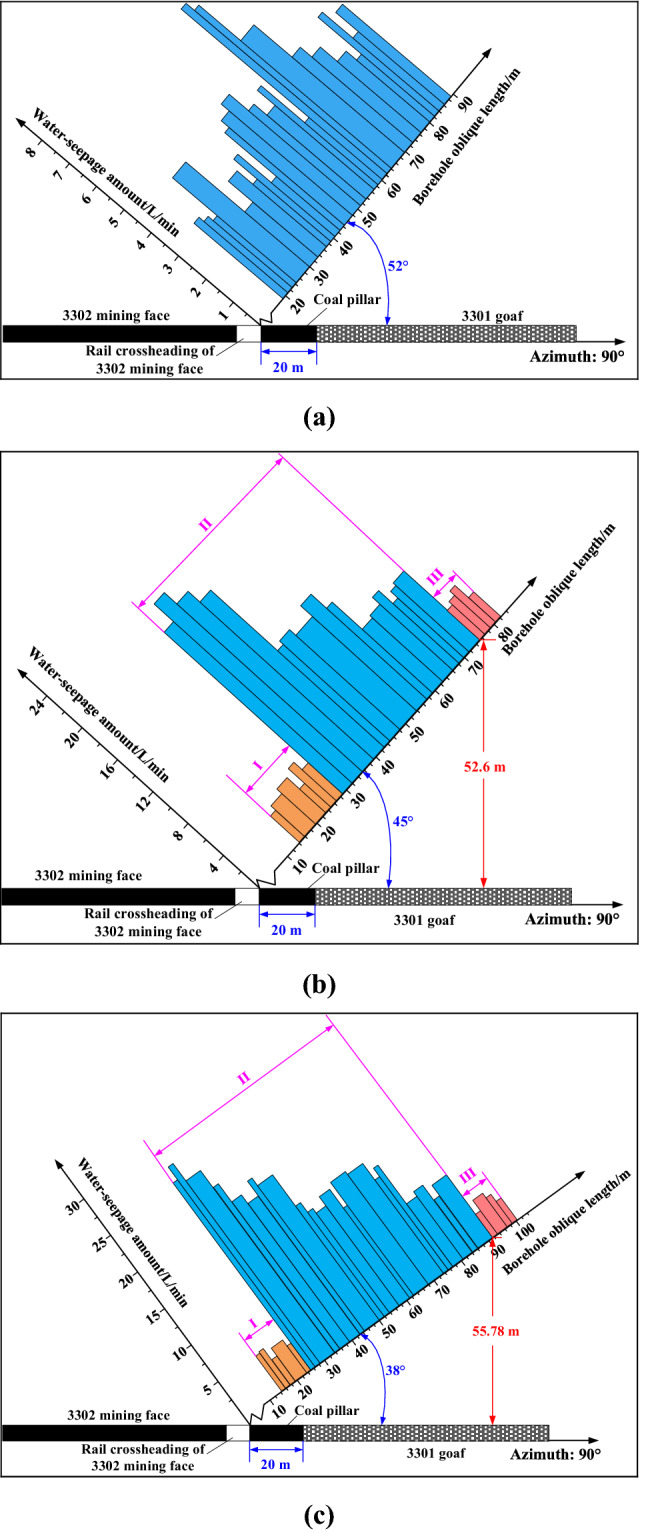
Water seepage histogram: (**a**) Borehole B1, (**b**) Borehole B2, and (**c**) Borehole B3.

The detection result of borehole B1 is presented in Fig. 10a. The integral overlying strata are unaffected by the mining, and the water injection in this borehole mainly seeps into the pre-existing fissures. Therefore, the water seepage in this borehole is low and the water seepage variation is non-significant. The water seepage is the lowest in the oblique length range of 36.2– 38 m, which is 2.46 L/min, and the largest in 59.6–61.4 m with 7.33 L/min. Comprehensively, the average water seepage in this borehole is 4.07 L/min.

The detection result of borehole B2 is presented in Fig. 10b. The water seepage is divided into 3 segments along the borehole oblique length, as described in the following procedures:

Segment I: This borehole segment is located above the coal pillar. Correspondingly, the overlying strata are unaffected by the mining activity, and the water injection in borehole mainly seeps into the pre-existing fractures as well. The water seepage is basically the same as that of borehole B1, and the values are all below 5 L/min. This phenomenon is presented in the oblique length range of 36.2–38 m.

Segment II: Compared with segment I, the water seepage increased significantly. Among them, the increase of water seepage is the highest in the oblique length range of 29–42 m, which is generally about 19.8–22.7 L/min, indicating that the borehole has penetrated into the fractured zone at this time. However, the water seepage decreases along the borehole, its value is still generally higher than that of borehole B1 or borehole B2 in the segment I, which indicates that the whole segment II is located in the WPFZ. This phenomenon is presented in the oblique length range of 29–74.4 m.

Segment III: The water seepage generally decreases to less than 5 L/min, indicating that the water permeability of this segment is unsmooth and the borehole has penetrated the WPFZ. This phenomenon is presented in the oblique length range of more than 74.4 m.

According to the detection result of borehole B2, the water seepage variation is the most obvious at the borehole oblique length of 74.4 m (i.e., the vertical height is 52.6 m), which is the demarcation point. Therefore, it can be concluded that the WPFZ height detected by borehole B2 is 52.6 m.

The detection result of borehole B3 is presented in Fig. 10c. Similarly, there is a significant demarcation point of water seepage at the borehole oblique depth of 90 m. Beyond the demarcation point, the water seepage is basically the same as that of borehole B1, indicating that the borehole may be located outside the WPFZ or inside the bending zone. Therefore, it can be concluded that the WPFZ height detected by borehole B3 is 55.8 m.

To sum up, when the overlying strata are not affected by mining, the water seepage is low. However, a large number of fractures as water channels are generated in the overburden affected by mining. Ultimately, the comprehensive detected results indicate that the WPFZ height is approximately 52.6–55.8 m.

##### Borehole camera method

(1) Detection scheme

**Figure 11:**
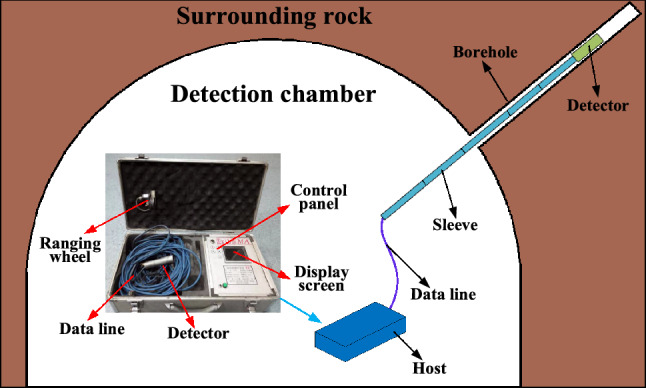
Detection scheme and equipment (This figure is drawn by the Microsoft office 2016, https://www.microsoft.com/zh-cn/microsoft-365/previous-versions/microsoft-office-2016).

In order to accurately determine the WPFZ height, the field detection follows the principle of one borehole for multiple purposes. On the basis of quantitative detection, the overlying strata fractures are observed directly by Panoramic Borehole Camera. Through borehole imaging, the development of rock fractures in the borehole is directly displayed in the form of images^34–37^. Borehole B3 is selected for this borehole camera detection, the detection scheme and equipment are present in Fig. 11. Panoramic Borehole Camera consists of a detector, sleeves, data lines, a ranging wheel, and a host. During the observation, the detector is slowly pushed through the sleeves to the top of the borehole. And then, the video along the borehole could be recorded and transmitted to the host in real time. The development of fractures in the overburden could be acquired accurately. Thus, the WPFZ height could be eventually determined according to the development and fragmentation of rock fractures.

(2) Detection results

**Figure 12:**
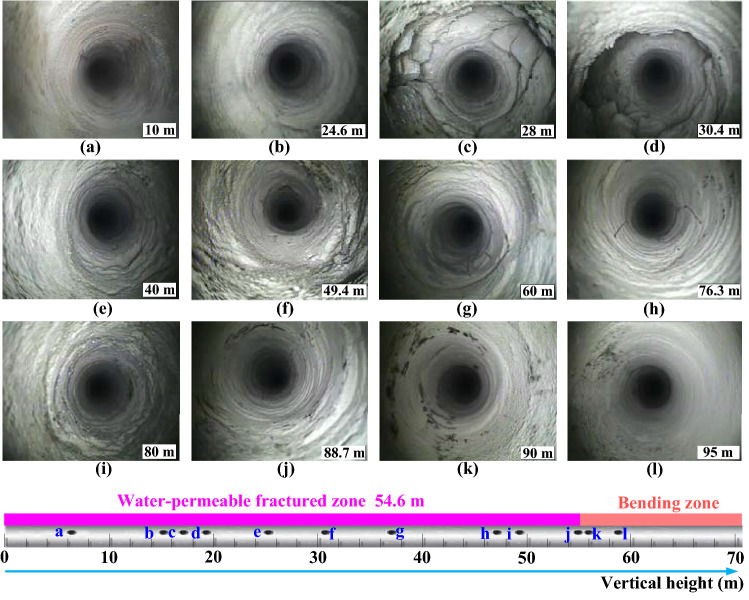
Borehole camera images and corresponding WPFZ range.

The images in Fig. 12 present the development of fractures in the borehole B3, and the length in the bottom right-hand corner of the images indicate the borehole oblique length. The borehole segment with an oblique length of less than 24.6 m is located above the coal pillar. The strata in this segment are not affected by mining, and the strata are complete without cracks, so the borehole wall is smooth and flat, as presented in Fig. 12a and b. However, the borehole segment with an oblique length of more than 24.6 m is affected by mining significantly. As presented in Fig. 12c and d, especially in the oblique length range of 24.6–30.4 m, the rock strata are damaged severely. There are a large number of criss-crossing fractures and compacted rock blocks. Additionally, the collapse of the borehole is even observed. According to these observation results, the roof separated and collapsed in the range of this borehole. As the observation continues, Fig. 12e and f present that the overburden is still broken in the oblique length range of 30.4–49.4 m, but the damage degree is lighter than that of the previous borehole segment. A large number of fractures are formed due to the settlement of the strata in this settlement, resulting in the discontinuity of the strata. As presented in Fig. 12g and h, the damage degree of overlying strata continues to lighten, the identification of the strike and width of fractures becomes distinct in the oblique length range of 49.4–76.3 m. In the oblique length range of 76.3–88.7 m, as presented in Fig. 12i and j, the fractures are mainly characterized by bedding, with a decrease in number and width. By inference, the strata move slightly and some tiny separations are generated in this range. When the oblique length range of borehole is more than 88.7 m, the borehole shape is consistent with that of the initial segment. As presented in Fig. 12k and l, the borehole wall without fractures is smooth and complete, and the rock strata in this segment don´t sink and break.

Correspondingly, the range of WPFZ is presented beneath the images. It can be seen that the oblique length of the borehole is 88.7 m, that is, the vertical length is 54.6 m, which is the boundary of the WPFZ, from which the overburden enters into the outer part of the WPFZ and the inner part of the bending zone. Through the same borehole B3, the WPFZ height determined by borehole camera method is 54.6 m, while that determined by downhole segmented water injection method is 55.8 m, with an error of only 2%. Thus, it can be found easily that the research results are highly consistent, which verifies the reliability of the in situ detections.

## Discussion

Based on the movement synchronous movement in overlying strata, a new method of OSSM is proposed to predict the WPFZ height. The method of OSSM is theoretical method. Compared with other methods, when it is applied, the grouping, lithology and thickness of overlying strata in the study area are necessary data, but these can be queried in the basic data of the mine. Therefore, the application of OSSM is convenient and cost-effective. There are many methods to predict WPFZ height, but each method has its own characteristics. Therefore, in order to choose the most advantageous method, the data reliability, operability and practicability of these methods need to be compared and evaluated.

### Evaluation of data reliability

The WPFZ height is predicted by three different methods in this study, we calculate the WPFZ height by empirical formula of 3301 face in this part as well. Borehole detection method is the most reliable method to determine the WPFZ height at present^38^. Through above researches, the detection results of borehole camera method and downhole segmented water injection method are relatively close. On the premise of maximum consideration of on-site safety, in order to facilitate comparative analysis, the highest prediction data of downhole segmented water injection method is selected as the benchmark. The comparison of WPFZ height obtained by each prediction method is presented in Table 3. As we can see, the WPFZ heights obtained by borehole camera method and the OSSM are 2.15% and 6.81% different from the benchmark respectively. The difference between the three heights is quite minute, which indicates the reliability of the research results in this study. Furthermore, the accuracy of the OSSM is fully verified as well. However, due to some factors, such as shallow buried depth, thin bedrock and thick alluvium are ignored, the height obtained by the empirical formula is quite different from the benchmark. Therefore, the traditional empirical formulas are not suitable for WPFZ height prediction in some specific geological environments.

**Table 3:** Comparison of WPFZ height obtained by different methods.

Method	WPFZ height/m	Error/%
Empirical formula method	35.0	37.28
OSSM	59.6	6.81
Borehole camera method	54.6	2.15
Downhole segmented water injection method	52.6–55.8	–

### Evaluation of operability and practicability

The above exploration presents that each prediction method applied in this paper can predict the WPFZ height accurately, but the operability and practicability are various for each method.

The relative size characteristics of fractures in overlying strata can be accurately obtained by downhole segmented water injection method, but the development shape of fractures can´t be observed. The sealing effect of the two capsules needs to be guaranteed in the field detection, and the construction details need to be paid attention to. In addition, the water injection probe needs to be pushed into the boreholes by a drilling rig, and the high-pressure water and gas need to be connected. Therefore, the workload of this method is strenuous.

The borehole camera method can visually observe the development location and shape of the fractures, and the WPFZ height detected by this method is the most accurate. However, this method can´t accurately compare the size of the fractures between the borehole walls. Additionally, there are many unavoidable problems during observation, such as the detector stuck by broken rocks or obscured by mud and so on, which needs to be detected many times.

Obviously, OSSM is a theoretical method, which can predict the WPFZ height easily. Compared with other methods, although the application of OSSM requires a comprehensive grasp of the geological and mining parameters, these are the basic parameters of mine production and can be easily queried. Therefore, the application of OSSM is convenient and cost-effective.

To sum up, the prediction of WPFZ height should not solely rely on conventional rules or empirical formulas. Reliable research methods should be selected and implemented only after the geological conditions and mining conditions are adequately emphasized, which provides scientific design basis for safe mining.

## Conclusions

In this study, the formation factors affecting the WPFZ height are demonstrated comprehensively by scaling model experiment. Through the experimental phenomenon, the WPFZ height is closely related to the movement characteristics of key strata and its follow-up strata. According to this, the new method of OSSM is established to predict the WPFZ height, which is based on the synchronous movement in overlying strata.

According to the calculation of OSSM, a reasonable WPFZ height range in 3301 mining face of Zhujiamao Coal Mine is predicted to be 59.6 m. The WPFZ height detected by downhole segmented water injection method is 52.6 m to 55.8 m, while that detected by borehole camera method is 54.6 m. These heights are similar to that calculated by OSSM, which indicates the reliability of the new method. Based on comprehensive consideration, the WPFZ height is finally determined as 52.6– 59.6 m in 3301 mining face.

Various methods for determining the WPFZ height are evaluated. Borehole detection method is the most reliable method to determine the WPFZ height at present. However, the workload of borehole detection method is strenuous. The OSSM proposed in this paper is characterized by simplification and higher accuracy.

In the future, the method of OSSM should be applied in more suitable mines to further verify its applicability and accuracy. Incidentally, this method should be further improved and optimized to reduce the application requirements and be more widely applied.
